# Application of DArT seq derived SNP tags for comparative genome analysis in fishes; An alternative pipeline using sequence data from a non-traditional model species, *Macquaria ambigua*

**DOI:** 10.1371/journal.pone.0226365

**Published:** 2019-12-12

**Authors:** Foyez Shams, Fiona Dyer, Ross Thompson, Richard P. Duncan, Jason D. Thiem, Andrzej Kilian, Tariq Ezaz

**Affiliations:** 1 Institute for Applied Ecology, University of Canberra, Canberra, Australian Capital Territory, Australia; 2 Centre for Applied Water Science, University of Canberra, Canberra, Australian Capital Territory, Australia; 3 Department of Primary Industries, Narrandera Fisheries Centre, Narrandera, New South Wales, Australia; 4 Diversity Arrays Technology, Bruce, Australian Capital Territory, Australia; University of Leeds, UNITED KINGDOM

## Abstract

Bi-allelic Single Nucleotide Polymorphism (SNP) markers are widely used in population genetic studies. In most studies, sequences either side of the SNPs remain unused, although these sequences contain information beyond that used in population genetic studies. In this study, we show how these sequence tags either side of a single nucleotide polymorphism can be used for comparative genome analysis. We used DArTseq (Diversity Array Technology) derived SNP data for a non-model Australian native freshwater fish, *Macquaria ambigua*, to identify genes linked to SNP associated sequence tags, and to discover homologies with evolutionarily conserved genes and genomic regions. We concatenated 6,776 SNP sequence tags to create a hypothetical genome (representing 0.1–0.3% of the actual genome), which we used to find sequence homologies with 12 model fish species using the Ensembl genome browser with stringent filtering parameters. We identified sequence homologies for 17 evolutionarily conserved genes (*cd9b*, *plk2b*, *rhot1b*, *sh3pxd2aa*, *si*:*ch211-148f13*.*1*, *si*:*dkey-166d12*.*2*, *zgc*:*66447*, *atp8a2*, *clvs2*, *lyst*, *mkln1*, *mnd1*, *piga*, *pik3ca*, *plagl2*, *rnf6*, *sec63*) along with an ancestral evolutionarily conserved syntenic block (euteleostomi Block_210). Our analysis also revealed repetitive sequences covering approximately 12% of the hypothetical genome where DNA transposon, LTR and non-LTR retrotransposons were most abundant. A hierarchical pattern of the number of sequence homologies with phylogenetically close species validated the approach for repeatability. This new approach of using SNP associated sequence tags for comparative genome analysis may provide insight into the genome evolution of non-model species where whole genome sequences are unavailable.

## Introduction

In recent years, advances in next generation sequencing technology have yielded higher resolution data for molecular genetic analyses. Sequencing data, ranging from short genomic fragments to whole genome sequencing, has been used to answer critical questions about evolutionary genetics using comparative genome analysis [1–3]. While whole genome sequencing provides the highest resolution for comparative analyses, it remains expensive and may not be cost effective for non-model species. Sequencing short genomic fragments, including molecular markers (microsatellites, SNPs) costs less and, while providing a lower resolution, may be useful for comparative genome analysis [4]. Bi-allelic Single Nucleotide Polymorphism (SNP) markers are widely used in population genetic studies. In most studies, sequenced data either side of the SNPs remain unused, although these sequences contain information beyond that used in population genetic studies, such as identification of evolutionarily conserved regions [5–7].

Diversity Array Technology (DArTseq^™^) produces restriction site-associated SNP markers using a combination of a complexity reduction method and a next generation sequencing platform [8]. Conceptually similar to double digest RADseq [4], DArTseq screens thousands of SNP markers across the genome, usually 69bp sequences (complexity reduction) containing a single nucleotide polymorphism in two alleles for a certain locus [8]. While a greater number of markers provides higher resolution for population genetic studies in plants [9] and in animals [10], low genome coverage (approximately 0.1–0.3%) and a lack of prior information about gene associations make these markers unappealing for comparative genomic studies. For example, the random sampling of such short sequence tags containing SNPs do not provide the exact location of each sequence in the genome, although there is always a chance that sequences are associated with particular genes.

In this study we used 6,776 DArTseq derived 69 bp sequences containing SNPs (STAGs) from an Australian native freshwater fish *Macquaria ambigua* (Richardson 1845) commonly known as golden perch. Here, we define a STAG as a single trimmed sequence (remaining product of a single 69 bp DArT marker after removing the restriction site-associated adapter) usually 69 bp or less in length where the polymorphic nucleotide is replaced with a standard ambiguity code. Hence, for more than one sample data set the replaced nucleotide with a standard ambiguity code determines polymorphism between individuals while the remainder of the 68 bp (or less) remains the same for the entire data set. The set of sequenced data (6,776 markers) was generated from 90 *M*. *ambigua* with an aim to perform population genetic analysis. Usually in population genetic studies with SNP markers, the single polymorphic nucleotide aids as a data point while the remaining 68 bp remain unused. We concatenated a total of 6,776 STAGs from 90 individuals of *M*. *ambigua* and used the concatenated sequence as a virtual low coverage genome for comparative genome analysis to identify evolutionarily conserved regions including genes. We compared this low coverage virtual genome with genomes from 12 fish species *Poecilia formosa*, *Astyanax mexicanus*, *Xiphophorus maculatus*, *Lepisosteus oculatus*, *Danio rerio*, *Oreochromis niloticus*, *Gadus morhua*, *Takifugu rubripes*, *Oryzias latipes*, *Gasterosteus aculeatus*, *Tetraodon nigroviridis* and *Latimeria chalumnae*. The specific aim of this study is to assign *M*. *ambigua* STAGs to specific genes and use them to identify evolutionarily conserved regions. Our study provides an alternate approach to identify the association of STAGs with a suite of evolutionarily conserved genes and genomic regions and highlights how STAGs can be used for comparative genome analysis to gain insight into genome evolution in non-traditional model species where whole genome sequences are unavailable.

## Materials and methods

### Animals and tissue collection

Fin clip and muscle tissues were collected from 90 *M*. *ambigua*. Fish were captured using boat-electrofishing from the Lachlan River, New South Wales, between Wallanthery (-33.34317688, 145.8420574) and Hillston (-33.47784, 145.52667) (7.5 kW Smith-Root model GPP 7.5 H/L boat mounted electrofishing unit). Tissues were stored in 95% ethanol. All collection procedures were performed by approved animal ethics protocol; University of Canberra Animal ethics project ID AEC 17–18, Fisheries NSW Animal Care and Ethics permit 14/10 and Scientific Collection Permit P01/0059(A)-2.0.

### Genomic DNA extraction and genotyping by sequencing

DNA extraction was performed using protocols developed by Diversity Array Technology Pty Ltd (DArTseq^™^) [8]. The quality of genomic DNA was evaluated by running agarose gel electrophoresis (1.2% agarose). Genotyping by sequencing was performed by DArTseq^™^ using a combination of DArT complexity reduction methods and next generation sequencing following protocols described in [8, 11–14]. Markers with a high call ratio (threshold 0.25) were filtered based on their polymorphic information content (>0.025) using a software package developed by DArT PL (http://www.diversityarrays.com/software.html). Here, a high call ratio represents the least proportion of missing values (to call both alleles of a marker) caused by sequencing error or low-quality genomic DNA.

### Low coverage genome construction, repeat masking and BLAST/BLAT analysis

We pooled and concatenated 6,776 STAGs from 90 individuals to generate a hypothetical low coverage genome of *M*. *ambigua* (Golden perch). The golden perch hypothetical genome (GP-H-Genome) was constructed in two steps: preparation of a hypothetical genome for each individual through concatenating all STAGs followed by a multiple alignment of all hypothetical genomes using software geneious (S1 Fig) [15]. To retain the same order of sequences for every individual during concatenation we arranged the markers ascending to their allele ID (unique marker ID) prior to generating an individual fasta file. We applied the following criteria for multiple alignment: global alignment with free end gaps along with a default multiple alignment setting including 65% similarity (5.0/-4.0), cost matrix, gap open penalty (GOP) - 12, Gap extension penalty (GEP)– 3 and Refinement iteration (RI)– 2. Pooling STAGs from 90 individuals captured missing alleles, providing improved coverage of the concatenated genome, and ensuring the most common nucleotide in the SNP position of a marker through multiple alignment.

We used “Repbase” to analyse the repetitive sequences of GP-H-Genome [16] and the Ensembl BLAST/BLAT (http://asia.ensembl.org/Multi/Tools/Blast?db=core) tool to identify sequence homologies against 12 available fish species (20/10/2017) [17]. While NCBI BLAST tools (https://blast.ncbi.nlm.nih.gov/Blast.cgi) are more popular and can provide a higher number of species to search against, we selected Ensembl over NCBI given its ability to perform BLAST and BLAT (BLAST Like Alignment Tool) at once, thus enabling identification of homologies with short sequences. We performed a normal sensitivity BLAST/BLAT search with 1 for a match score and -3 for a single mismatch score, along with enabled repeat masking and filtering low complexity regions. We restricted the number of hits with a threshold maximum E-value of 1e^-10^.

Despite the benefits of the concatenation approach, including ease of handling and lower computational time, a possible drawback is the probability of getting false positive hits relative to an individual BLAST search of each STAG. We characterised a hit as a false positive if it fulfilled three criteria: (i) the sequence length was greater than 69 bp, (ii) the sequence starts with the restriction fragment (Contains TGCAG at the beginning) at the 5’ end and (iii) the sequence contains the whole or a concatenated part of the restriction fragment (TGCAG). As DArT PL used the Pst*I* restriction enzyme (best scored out of four pairs; refer to methods) as a 5’end anchor, all markers had a common 5’ end “TGCAG”. Therefore, a false positive hit must contain two sets of such sequences or one complete sequence at the beginning and a part at the end. Only the first nucleotide “T” can randomly occur at the end of the sequence (with probability 1 in 4). We excluded such sequences being identified as a false positive. We also excluded homologies with concatenated first two nucleotides “TG” from being identified as false positive hit probability of occurrence (1 in 16) and the abundant di-nucleotide repeats (AC, TG, CT) described in the results section. As the chance of three or more nucleotides of the restriction fragments randomly occurring is low (TGC- 1 in 64, TGCA– 1 in 256, TGCAG– 1 in 1024) we excluded these hits (approximately 1.42%, S2 Fig) from the analysis to avoid potential error. We also randomly selected 600 STAGs (approximately 10% of the total set) and BLAST them against the *O*. *niloticus* (Nile tilapia) genome as individual alleles (without concatenation) for further validation.

### Identification of evolutionarily conserved genes

**Fig 1:**
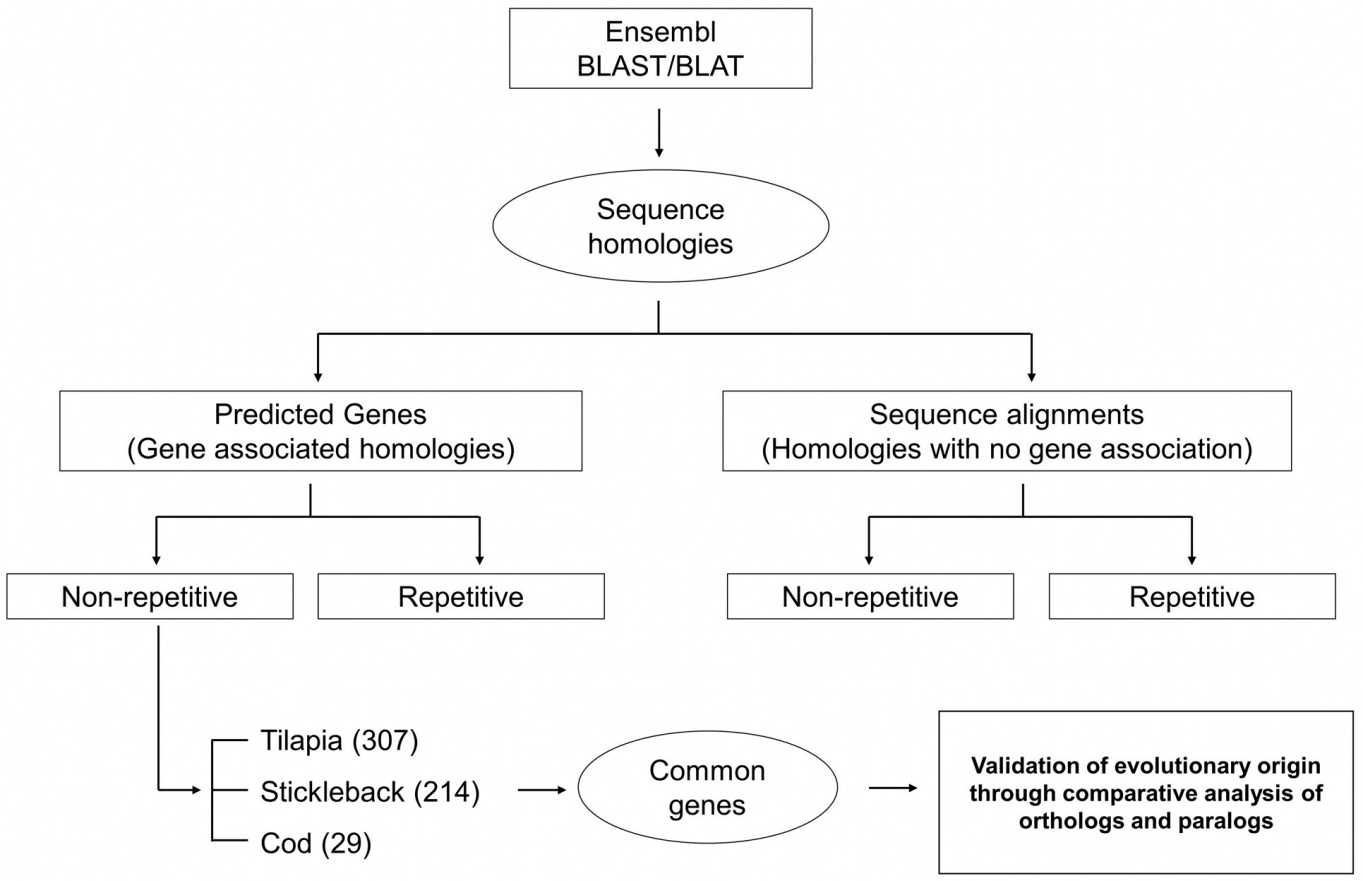
A schematic diagram of selecting evolutionarily conserved genes. Numbers against each species represents predicted genes resulted from non-repetitive sequence (STAGs) homologies.

Evolutionarily conserved genes were selected from gene associated sequence homologies (orthologs) based on our cut-off E-value (1e^-10^). We performed this analysis in two steps; first, selecting common genes within multiple species and second, validating the origin of genes using online databases, such as Genomicus (http://www.genomicus.biologie.ens.fr/genomicus-95.01/cgi-bin/search.pl). A schematic representation of these steps is presented in Fig 1. From the available fish species, we selected three species based on several criteria: i) phylogenetic relationship, ii) diverse habitat and, iii) number of homologies (including unique STAGs) obtained from the BLAST/BLAT search. Based on these criteria we selected a freshwater fish (*O*. *niloticus*; Nile tilapia), an anadromous fish (*Gasterosteus aculeatus*; three spined- stickleback) and a marine fish (*Gadus morhua*; Atlantic cod) for this analysis. Common genes with non-repetitive sequence homologies were selected for further analysis as evolutionarily conserved genes. Genomicus tools [18] were used to interpret evolutionary origins of conserved genes and synteny through isolation of orthologs and paralogs within fishes as well as within vertebrates.

## Results

### *M*. *ambigua* genomic representation

A total of 6,776 STAGs were scored for 90 *M*. *ambigua* after screening with an average read depth threshold (>60 per locus). The multiple alignment resulted in a 405,803 bp consensus sequence with 47.5% GC content, which is our hypothetical genome for golden perch (the GP-H-Genome).

### Masked repetitive sequences

Analysis of masked repetitive sequences revealed a total of 628 repetitive fragments representing 49,561 bp covering approximately 12.21% of the GP-H-Genome (Table 1). Approximately 95% of the repetitive sequences were transposable elements of three categories: DNA transposons (51.7%), endogenous retroviruses (3.9%) and retrotransposons (44.4%). The retrotransposons were almost equally divided into LTR retrotransposons (54%) and Non-LTR retrotransposons (46%) (Table 1). In addition to transposable elements, 3 repetitive fragments aligned with Caulimovirus.

**Table 1:** Major classes of repetitive sequences in GP-H-Genome (the concatenated *M*. *ambigua* genome covering approximately 0.1–0.3% of the whole genome).

Repeat class	Number of hits	Total Length of all alignment (bp)	Pseudogene
Integrated virus	3	131	-
Interspersed repeat	10	825	-
DNA transposon	300	24,405	1
Endogenous Retrovirus	27	1,829	-
LTR Retrotransposon	141	11,414	1
Non-LTR Retrotransposon	139	9,526	-
Simple repeat	8	1,431	-
**Total**	**628**	**49,561**	**2**
**Proportion**		**12.2%**	

### Sequence homologies (Ensembl BLAST/BLAT hits)

Ensembl BLAST/BLAT search of GP-H-Genome identified a range of sequence alignments homologous (includes both ortholog and paralog) to 12 fish genomes (Table 2). The least number of homologies (23) were identified against the *L*. *oculatus* (Spotted gar) genome while the highest number (1491) were identified against *Gadus morhua* (Atlantic Cod) (Table 2).

**Table 2:** Ensembl nucleotide BLAST analysis hits. Major groups of homologies where non-repeat alignments are sequence homologies without any association with genes. Repetitive alignments are the homologies of full or partially repetitive sequences. And Genes represent sequence homology both repetitive and non-repetitive have association with at least one gene. The proportion of GP-H-Genome has homology with a certain species is shown as %.

Species	Non-repeat alignments			Repetitive alignments			Genes			Total			% of GP-H-Genome
	Number of hits	GP fragments	length (bp)	Number of hits	GP fragments	length (bp)	Number of hits	GP fragments	length (bp)	Number of hits	GP fragments	length (bp)	
*Poecilia formosa*	66	51	3228	72	29	1606	246	140	8889	384	220	13723	3.38
*Astyanax mexicanus*	3	3	208	48	28	1406	49	31	1770	100	62	3384	0.83
*Gadus morhua*	9	9	573	1137	115	6498	345	93	5430	1491	217	12501	3.08
*Latimeria chalumnae*	0	0	0	173	38	2078	97	27	1471	270	65	3549	0.87
*Takifugu rubripes*	52	49	2954	143	45	2499	209	142	8932	404	236	14385	3.54
*Oryzias latipes*	46	45	2798	16	10	501	97	84	5354	159	139	8653	2.13
*Xiphophorus maculatus*	58	46	2893	28	20	1157	134	110	7044	220	176	11094	2.73
*Lepisosteus oculatus*	1	1	57	7	7	359	15	9	587	23	17	1003	0.25
*Gasterosteus aculeatus*	112	102	6077	163	63	3368	417	253	16052	692	418	25497	6.28
*Tetraodon nigroviridis*	40	39	2422	170	49	2677	205	131	7955	415	219	13054	3.22
*Oreochromis niloticus*	419	161	10289	111	38	2086	492	356	22423	1022	555	34798	8.57
*Danio rerio*	3	3	166	260	57	3234	527	83	4812	790	143	8212	2.02

**Fig 2:**
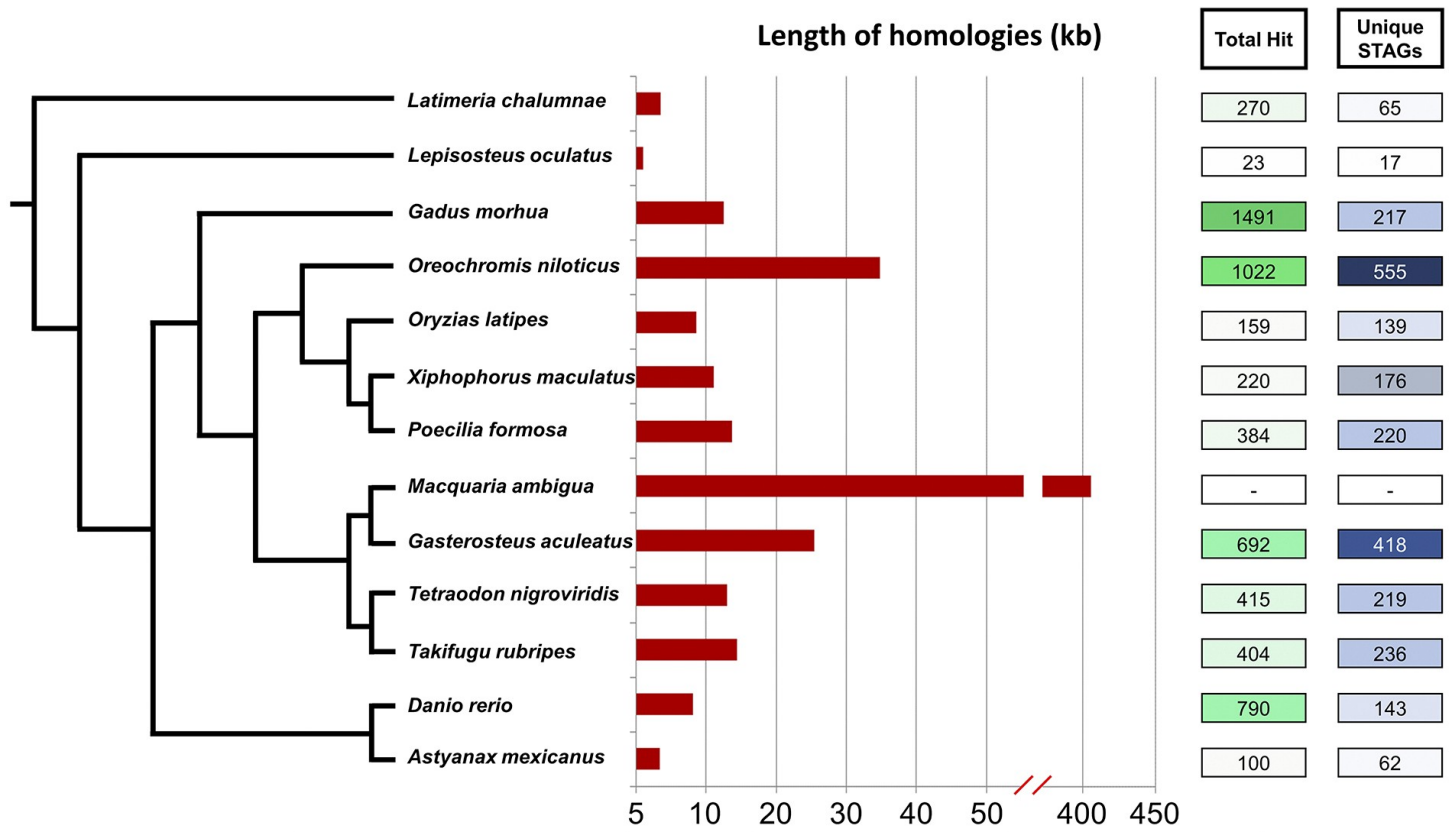
Ensembl Nucleotide BLAST/BLAT hits. Phylogeny (Not according to scale) adopted from Betancur-R et al. (2017) [19] represents *Macquaria ambigua* (golden perch) lineage with all reference fish used for the analysis. Length (in kilo-base pairs) represents the proportion of golden perch hypothetical genome (GP-H-Genome) homologous with 12 fish species (*M*. *ambigua* bar represent the length of the total GP-H-Genome). STAGs stand for a single trimmed sequence (remaining product of a single 69 bp DArT marker after removing the restriction site-associated adapter) usually 69 bp or less in length where the polymorphic nucleotide is replaced with a standard ambiguity code.

The number of gene associated homologies (ortholog/paralog) identified from Ensembl BLAST/BLAT analysis ranged between 15 (*L*. *oculatus*) to 527 (*D*. *rerio*). As gene associated homologies contains both repetitive and non-repetitive STAGs alignment, we found multiple genes association for few repetitive STAGs. The analysis revealed only di-nucleotide repeats (AC, TG, CT) among the repetitive homologies predominantly AC and TG repeats. Hence, even the highest number of gene associated homologies was identified against the *D*. *rerio* (527) genome, the number of GP-H-Genome fragments (unique STAGs) was comparatively low (83) (Fig 2). On the other hand, the highest and second highest number STAGs with gene association homologies were with *O*. *niloticus* (356) and *Gasterosteus aculeatus* (253), reflecting their close taxonomic relationship with *M*. *ambigua*. For greater accuracy we excluded all the repetitive STAGs alignment homologies from the analysis for evolutionarily conserved genes.

A comparison between GP-H-Genome BLAST/BLAT hit and individual BLAST/BLAT hit (e.g. linkage group, genomic location, length of the alignment, %ID) for a certain STAG present in GP-H-Genome (S1 Table) suggests no significant tendency to nominate the homologies as a false positive. Furthermore, the probability of getting a false positive hit due to concatenation of two adjacent markers is reduced by the pipeline and the algorithm used for Ensembl BLAST/BLAT, including percentage alignment and setting the E-value. For the present study, we used a low E-value threshold (1e^-10^) to minimise the chance that a sequence homology with more than 69 bp was a false positive hit. In comparing individual BLAST/BLAT and GP-H-Genome BLAST/BLAT searches, we found two homologies with 69+ bp in the concatenated genome (S1 Table), although none of them were a false positive as per the criteria mentioned above. Hence, having such hits (above 69) might be a beneficial property of the concatenation approach.

### Evolutionarily conserved genes

**Fig 3:**
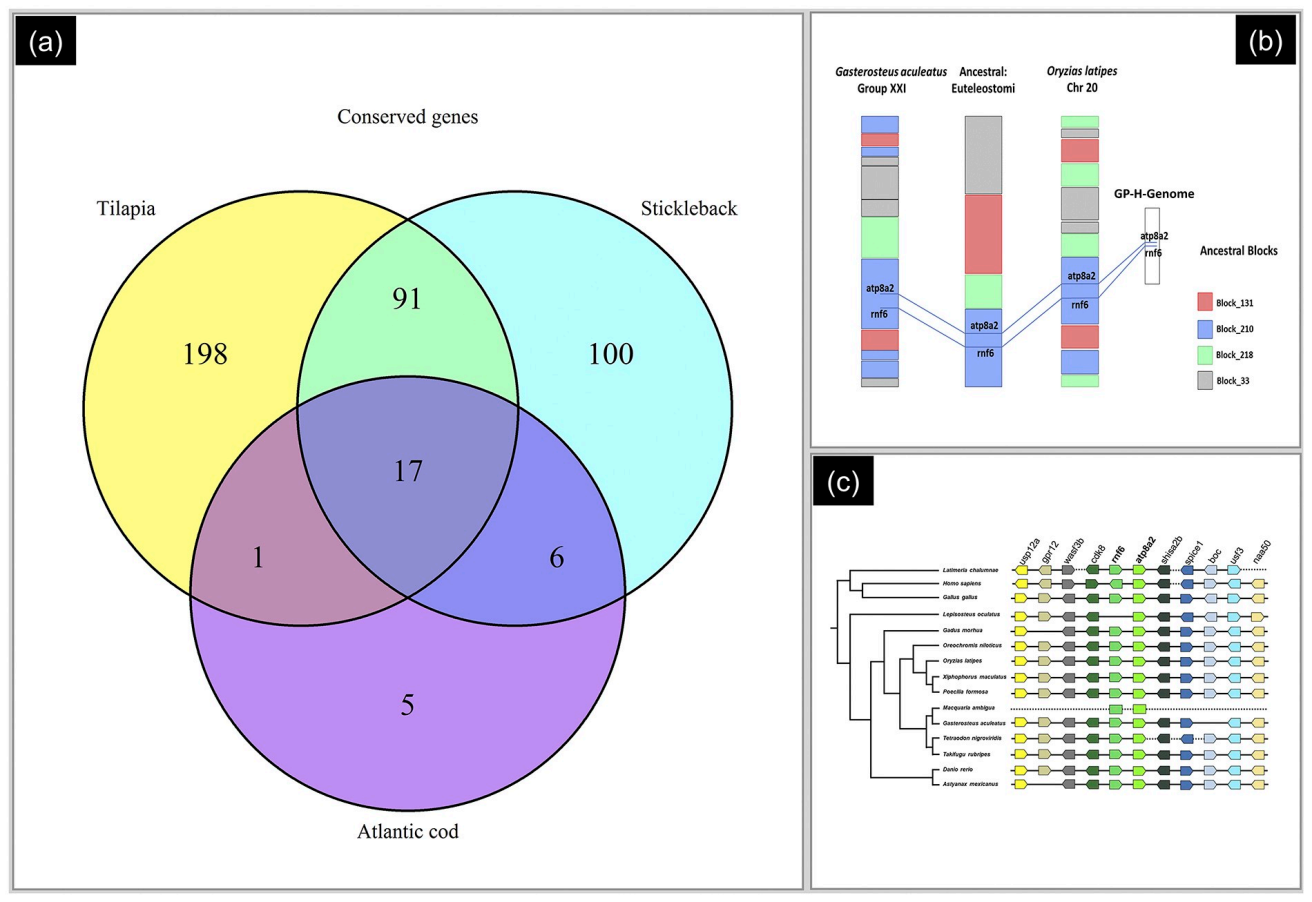
Evolutionarily conserved genes. (a) Number of genes have homologies with non-repetitive part of GP-H-Genome. Colour code represents the species against the BLAST hits obtained. 17 genes common in all three species. (b) Gene block arrangement. Comparison of conserve gene block arrangement for the region encompassing genes *atp8a2* and *nrf6* genes on *Gasterosteus aculeatus* Chromosome XXI and *Oryzias latipes* Chromosome 20 with the ancestral Euteleostomi blocks predicted in Genomicus database (c) *atp8a2* and *nrf6* genes are conserved compared to other vertebrate species. Arrows indicate direction of the transcription. Gene order and orientation is unknown for *M*. *ambigua*.

Using the criteria as described in the methods (Fig 1), we screened for gene associated homologies with the *O*. *niloticus*, *Gasterosteus aculeatus* and *Gadus morhua* genome. Filtering for false positive hits resulted 476 genes in *O*. *niloticus*, 409 genes in *Gasterosteus aculeatus* and 342 genes in *Gadus morhua*. As these gene-associated homologies are also composed of repetitive and non-repetitive STAGs in GP-H-Genome, we filtered out all genes selected from repetitive STAGs, resulting in 307 genes in *O*. *niloticus*, 214 in *Gasterosteus aculeatus* and 29 in *Gadus morhua*, respectively. We identified 17 evolutionarily conserved genes across the three species (Fig 3a) (S2 Table). These include 8 genes (*cd9b*, *pik3ca*, *plagl2*, *rhot1b*, *sh3pxd2aa*, *si*:*ch211-148f13*.*1*, *si*:*dkey-166d12*.*2*, *zgc*:*66447*) conserved across fish genera with ancestral roots dating back 550–420 MYA (S3 Table) [18] (http://www.genomicus.biologie.ens.fr/genomicus-94.01/cgi-bin/search.pl), and 9 genes (*atp8a2*, *clvs2*, *lyst*, *mkln1*, *mnd1*, *piga*, *plk2b*, *rnf6*, *sec63*) conserved across vertebrates with ancestral roots dating back to 1500 MYA.

The comparative orthology analysis of the 17 selected genes revealed that orthologues of *atp8a2*, *clvs2*, *lyst*, *mkln1*, *pik3ca*, *plagl2*, *rnf6* genes are conserved across all vertebrates while *mnd1*, *piga and sec63* are conserved beyond vertebrates (fungi/Metazoa group: Yeast Chr XV), while fish specific genes share either Craniata or Euteleostomi as a common ancestor. Analysis of linkage group associations revealed two vertebrate specific genes *atp8a2* and *rnf6* that share the same linkage group across all selected species (e.g. *Gasterosteus aculeatus* chromosome XXI, *O*. *latipes* Chromosome 20, *O*. *niloticus* scaffold 831157). Since this suggests that orthologues of *atp8a2* and *rnf6* are part of a conserved homologus syntenic block (HSBs) across all the vertebrate species, we investigated the association of other genes in the same syntenic block (Fig 3c). Conservation of the syntenic block across vertebrates (including human and chicken) was found for 10 genes associated with the reference gene, although one of the reference genes (*rnf6*) showed no homologies in *L*. *oculatus*.

## Discussion

Our study demonstrated that DArTSeq derived STAGs can be used for comparative genome analysis to infer genome evolution within teleost fishes. The mapping strategy used for the concatenated *M*. *ambigua* genome revealed a variable number of homologies across fish species (maximum: 555 STAGs of GP-H-Genome with *O*. *niloticus*; minimum: 17 STAGs of GP-H-Genome with *L*. *oculatus*), leading to a hierarchical pattern supporting the phylogenetic position of the taxa in the fish tree (Fig 2). Furthermore, the percentage of repetitive sequences (structurally similar to microsatellites), along with protein coding genes, was similar to that achieved by whole genome sequencing approaches [20] or even sequencing of certain genomic blocks (e.g. chromosome) [1].

The concatenated *M*. *ambigua* genome represents random segments of the original genome. Our study revealed about 12% of these segments are repetitive elements (Table 1) predominantly LTR and Non-LTR retrotransposons. These transposable elements are abundant in the eukaryotic genome (around 50% in human) and play a vital role in epigenomic regulation [21]. Active transposable elements are highly mutagenic and often responsible for insertion and deletion in protein coding genes resulting in genome rearrangement and irregular recombination [21–23]. Transposable elements are also useful for comparative genomics across a range of species [24]. In this study we have only detected transposable elements associated with restriction sites (due to DArTseq pipeline). Considering the proportion of these transposable elements and their function, future population genetic studies associated with these elements might be useful for fish species from diverse habitats.

The abundance of repetitive-type gene associated homologies over non-repetitive types in other fish species (such as in *Gasterosteus aculeatus*, 203 gene associate repetitive homologies (48% of total gene associated homologies in *Gasterosteus aculeatus*) while in *Gadus morhua* almost 92% gene associated homologies were repetitive) also validates the taxonomic lineage of the study species. Although, the data set only contains partial repeats, this could be a result of the nature of DArT seq markers (restriction site-association) and the threshold E-value of the BLAST analysis that we set.

Along with an assessment for the false positive hits (S1 Table), which discards the probability of getting a false homology due to concatenation, the High-scoring Segment Pair (HSP) distribution (S3 Fig) produced by Ensembl BLAST also justifies the approach. The hierarchical pattern of horizontal distribution of hits (sequence homologies) with multiple species is complementary to their phylogenetic relationship. Furthermore, the number of sequence homologies along with unique GP-H-Genome fragments (Fig 2) further indicates the repeatability of the approach. Although having highest homologies with *Gadus morhua* seems contradictory, the HSP distribution and number of unique GP-H-Genome STAGs comes with a valid justification. One of the reasons might be the abundance of transposable elements in the genome of the species. In this study, we found the highest hit producing STAG (545 hits against one STAG) was a dinucleotide tandem repeat (TG)_n_.

The present study represents the first attempt to use the flanking regions of DArT seq derived Single Nucleotide Polymorphism (SNP) markers for comparative genome analysis. Although similar markers such as Restriction site-associated DNA (RAD) has been demonstrated useful for comparative genome analysis [25–27], the present study offers an alternative pipeline for such markers. Despite the successful demonstration for the potential use of the pipeline across multiple fish species, one possible drawback is its application to other groups of animals such as mammals, birds is yet unknown. Future studies focused on such groups are required to investigate the suitability of the technique.

### Ancestral syntenic Block

A total of 17 protein coding genes were identified through a stringent screening of gene associated homologies. Of these 17 common genes, eight are fish specific (S3 Table) with diverse cellular functions including disease association. For example, one of the fish specific genes *CD9*, with an ancestral root dating back to 420 million years, is involved in cell adhesion, cell motility and tumour metastasis and is essential for sperm-egg fusion. Another fish specific gene *plk2b* (ancestral root euteleostomi), plays a vital role in cell cycle progression. The remaining ten vertebrate specific genes are involved in multiple cellular functions (*atp8a2*: required for normal visual and auditory function, *mnd1*: required for proper homologous chromosome pairing and efficient cross-over and intragenic recombination during meiosis). Comparative ortholog and paralog analysis (reference species: *O*. *niloticus*) revealed all eight fish specific genes are paralogs due to a single or multiple (pik3ca) duplication event (mostly in ancestral Clupeocephala (300 MYA). Multi species comparisons revealed conservation of two genes (*atp8a2* and *rnf6*) in a certain syntenic blocks across *O*. *niloticus* (GL831157), *Gasterosteus aculeatus* (Group XXI) and *Gadus morhua* (Genescaffold 733) (S2 Table). Further analysis revealed that these two genes have been conserved for over 420 million years since the divergence of Euteleostomi. The Euteleostomi syntenic block 210 containing these two genes is conserved across all nodes of vertebrate speciation (Fig 3b), suggesting the potential use of STAGs for identification of evolutionarily conserved genes and syntenic blocks.

## Conclusion

The pattern of sequence homologies (hierarchical to the phylogenetically closest species), accumulation of abundant repetitive sequences, and evolutionarily conserved sequences associated with genes in the concatenated *M*. *ambigua* (golden perch) genome suggests that this concatenated genome provides a partial but valid representation of the complete genome of the species. Through identification of evolutionarily conserved ancestral blocks, our study has demonstrated that DArT seq derived STAGs can be used for more than population genetic studies. This offers an alternative pipeline for comparative genome analysis of species when the annotated genomic data is unavailable for non-model species. The present approach will assist similar studies in other non-traditional model species.

## Supporting information

S1 Fig A schematic diagram of low coverage genome construction. Panel 1: concatenating STAGs for each individual as a hypothetical contiguous sequence (shown as contig) considering standard ambiguity code; panel 2: multiple alignment of representative hypothetical contigs; panel 3: consensus sequence as partial genome of the representative population or species.
(TIF)

S2 Fig Assessment for the false positive hits. First pie (Left) represents the percentage of positive hits (98.58%) and false positive hits (1.42%). The second pie represents the types of false positive hits (number of nucleotides from the sequence “TGCAG”).
(TIF)

S3 Fig High-scoring segment pair (HSP) distribution. Each panel represent a species. GP-H-Genome represented with black and white striped line and red bars represent each homology. Horizontal distribution of red bar suggests for number of unique fragment homology and vertical distribution represents presence of multiple homologies against a single query sequence (STAG of GP-H-Genome).
(TIF)

S1 Table A comparison of BLAST search between concatenated hypothetical genome (GP-H-Genome) and individual STAGs. The first column represents the n^th^ SNP tag out of randomly selected 600 (approximately 10% of total) while the missing values has been excluded due to absence of homologies with *Oreochromis niloticus* genome under same criteria (threshold E-value: 1e^-10^).
(DOCX)

S2 Table GP-H-Genome fragments (STAGs) with maximum E-value for 17 evolutionarily conserved genes. Length of the homology varies from 57–72 predominantly 69 base pairs. Calculated score based on E-value, length and percentage of alignment also predominant with *Gasterosteus aculeatus* suggesting for a closest phylogenetic lineage.
(DOCX)

S3 Table Evolutionary conserved genes with Euteleostomi as a predominant root species. Gene *atp8a2* and *rnf6* are conserved to a specific syntenic block across all species (Chromosome 21 in *Gasterosteus aculeatus* while in *O*. *niloticus* and *Gadus morhua* consequently in scaffold GL831157 and GeneScaffold 733).
(DOCX)
