# Supplementary figures and images for: Application of DArT seq derived SNP tags for comparative genome analysis in fishes; An alternative pipeline using sequence data from a non-traditional model species, Macquaria ambigua
Source: PLoS One. 2019 Dec 12;14(12):e0226365. doi: 10.1371/journal.pone.0226365 (PMC6907852; doi:10.1371/journal.pone.0226365)

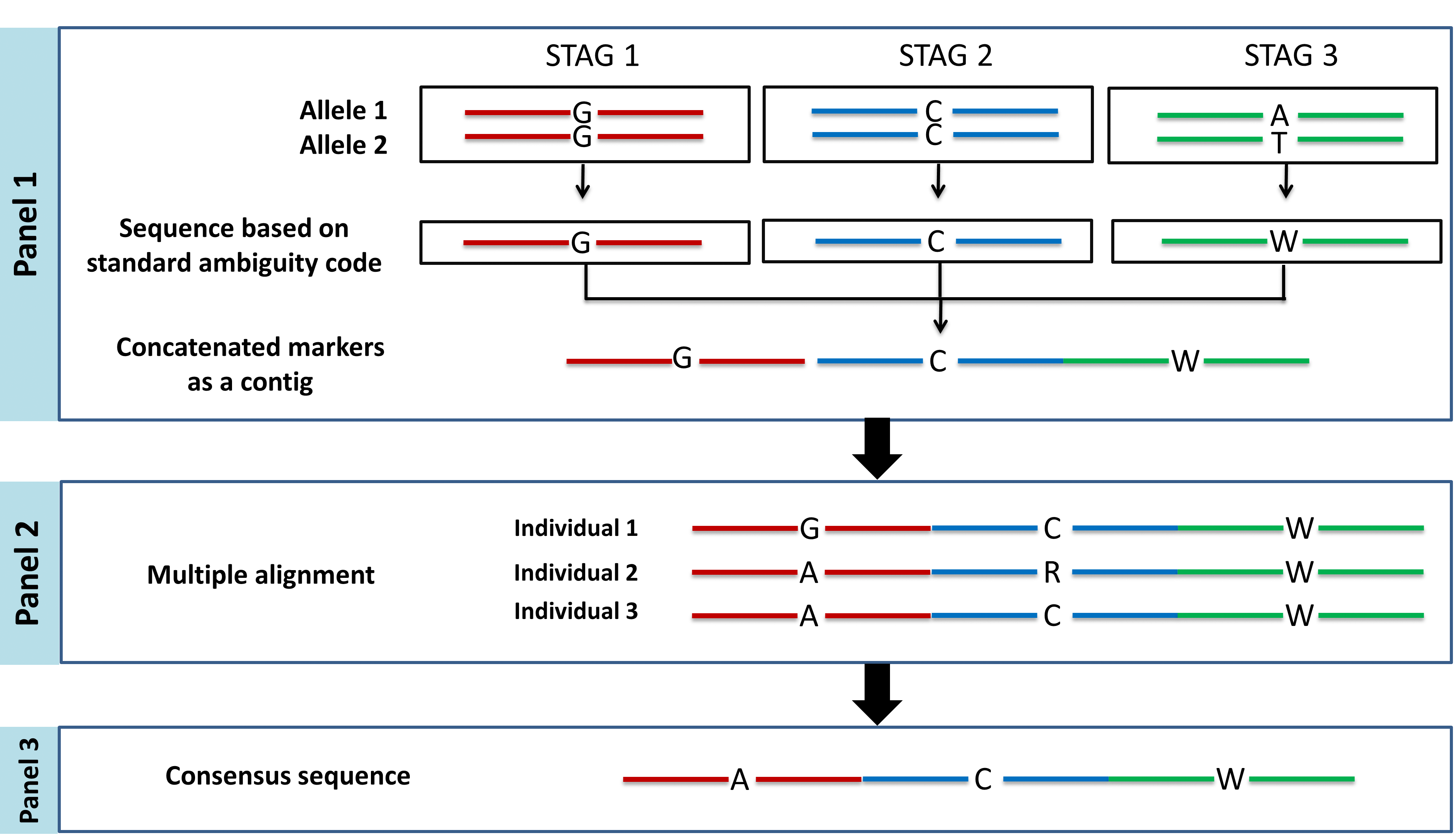

Supplement: S1 Fig — Panel 1: concatenating STAGs for each individual as a hypothetical contiguous sequence (shown as contig) considering standard ambiguity code; panel 2: multiple alignment of representative hypothetical contigs; panel 3: consensus sequence as partial genome of the representative population or species. (TIF) [file pone.0226365.s001.tif]

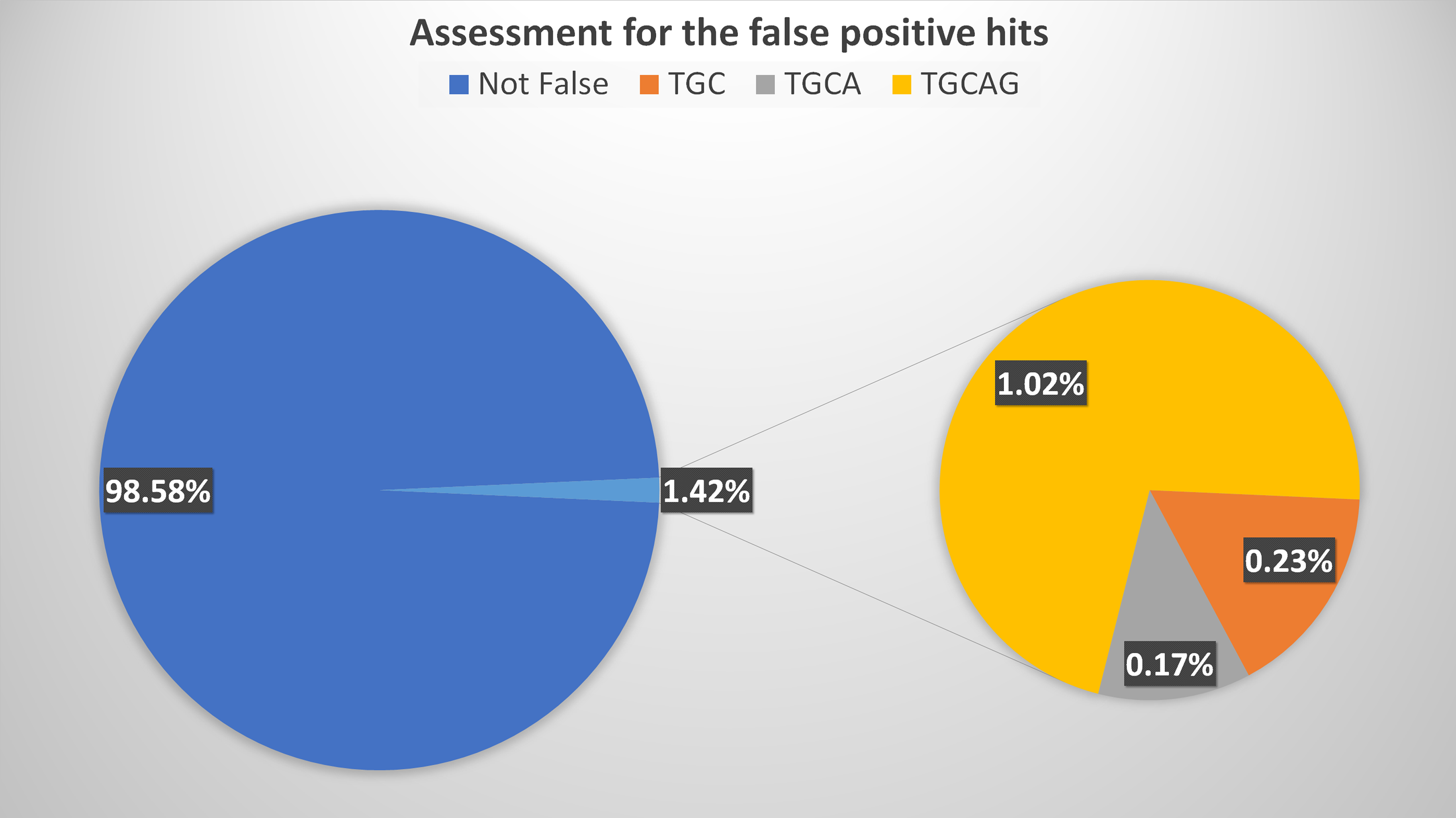

Supplement: S2 Fig — First pie (Left) represents the percentage of positive hits (98.58%) and false positive hits (1.42%). The second pie represents the types of false positive hits (number of nucleotides from the sequence “TGCAG”). (TIF) [file pone.0226365.s002.tif]

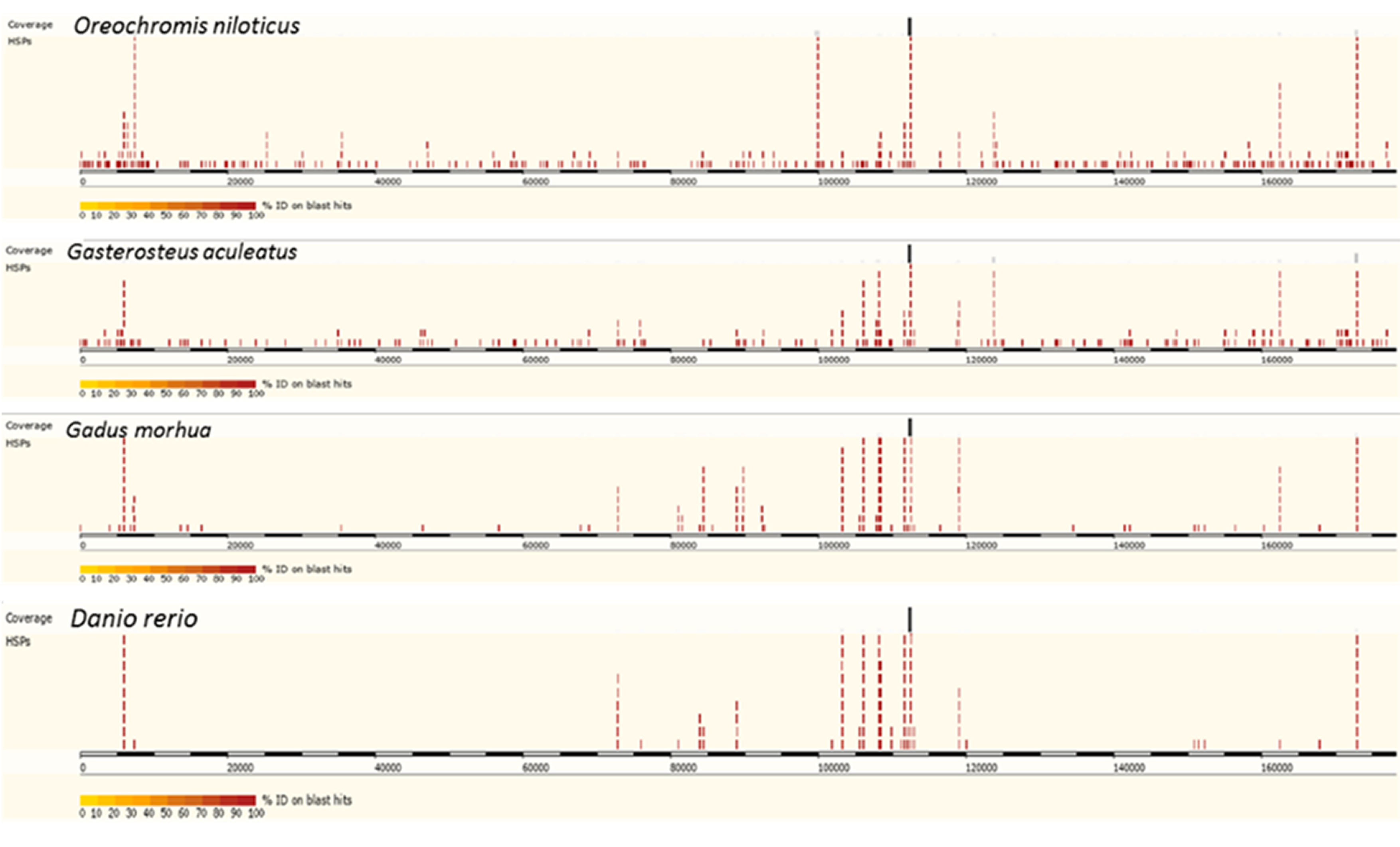

Supplement: S3 Fig — Each panel represent a species. GP-H-Genome represented with black and white striped line and red bars represent each homology. Horizontal distribution of red bar suggests for number of unique fragment homology and vertical distribution represents presence of multiple homologies against a single query sequence (STAG of GP-H-Genome). (TIF) [file pone.0226365.s003.tif]
