# Supplementary material for: Application of DArT seq derived SNP tags for comparative genome analysis in fishes; An alternative pipeline using sequence data from a non-traditional model species, Macquaria ambigua
Source: PLoS One. 2019 Dec 12;14(12):e0226365. doi: 10.1371/journal.pone.0226365 (PMC6907852; doi:10.1371/journal.pone.0226365)
Supplement: S1 Table — The first column represents the nth SNP tag out of randomly selected 600 (approximately 10% of total) while the missing values has been excluded due to absence of homologies with Oreochromis niloticus genome under same criteria (threshold E-value: 1e-10). (DOCX) [file pone.0226365.s004.docx]

**S1 Table. A comparison of BLAST search between concatenated hypothetical genome (GP-H-Genome) and individual STAGs.** The first column represents the n^th^ SNP tag out of randomly selected 600 (approximately 10% of total) while the missing values has been excluded due to absence of homologies with *Oreochromis niloticus* genome under same criteria (threshold E-value: 1e^-10^).

| **Sl (out of 600)** | **Sequence** | **Individual BLAST** | | | **GP-H-Genome BLAST** | | |
| --- | --- | --- | --- | --- | --- | --- | --- |
|  |  | Linkage group  (Genomic location) | Length  (bp) | Identity  (%) | Linkage group  (Genomic location) | Length | Identity  (%) |
| 3 | TGCAGACATTCGCACTGATCTCTCACATCCTCCATAATGCCATGTGTGCCTAGTTGACCAGATATTGCA | **GL831145** (4845937-4846004) | 68 | 92.65 | **GL831145** (4845937-4846004) | 68 | 92.65 |
| 4 | TGCAGATTATGTAGCAGCTGGATCATTAGCGTAGAGGGTCGAATGGCATG | **GL831197**  (185255-185304) | 50 | 96 | **GL831197**  (185255-185306) | 52 | 96.15 |
| 10 | TGCAGGAAATGTGCCTCTGGGTACACACTGACAGGGTCCAAGTGTTTGGGTAATTGCATAAAAGAGAAT | **GL831288** (1668443-1668495) | 53 | 96.23 | **GL831288** (1668443-1668495) | 53 | 96.23 |
| 11 | TGCAGTTCATTTGCTGATGAAAGGGGATTCATGCTCAATTATATCTTAATGGGTTGGAAATAACAGCAT | **GL831245** (1041491-1041559) | 69 | 95.65 | **GL831245** (1041491-1041559) | 69 | 95.65 |
| 14 | TGCAGAAAACGTTAGGTTTACCCAACTCAAACTGTCCCCAGAGCCTCAGCCAAAACAGCTGTCAGTGTG | **GL831226**  (470451-470519) | 69 | 98.55 | **GL831226**  (470451-470519) | 69 | 98.55 |
| 59 | TGCAGCCCTCTTTAATCTTATTCCTGTGGGTCTGAGAGTGGTGGCCATCCAGGGAGTGAAGAGTGGCTT | **GL831134** (7053704-7053772) | 69 | 97.1 | **GL831134** (7053703-7053773) | 71 | 97.18 |
| 78 | TGCAGGATCCCCAAAAACATGACCATCCCCAGTGAGGGACTGCCAGTGAAGGTTAGTCAGACTGGAGGT | **GL831152** (2944944-2944997) | 54 | 94.44 | **GL831152** (2944944-2944997) | 54 | 94.44 |
| 90 | TGCAGGTCTTATCACCCAGGGATGTTAAAGCTGAACCCTTGAGATATAAGAACATATCAGCACATTAAC | **GL831158** (2245349-2245417) | 69 | 89.86 | **GL831158** (2245349-2245417) | 69 | 89.86 |
| 91 | TGCAGGTGATGTACAACCAGTCCACAGCCACCACCTGCTTCCACAGTCTCCCACTGCTGCGCTGTGTGT | **GL831258** (1036314-1036382) | 69 | 94.2 | **GL831258** (1036313-1036383) | 71 | 94.37 |
| 94 | TGCAGTAGATGAAGGCTACTTAACAAGCTGCCTTAAGCTCTTCAGCAGGCTTTCTGTCAGCTACTGGGG | **GL831211** (2341799-2341866) | 68 | 97.06 | **GL831211** (2341799-2341866) | 68 | 97.06 |
| 127 | TGCAGAAGCACAAAAGATTGGATTCACTTCATCAAGTCAGACACATTACTCTCAACACAAACAAGATGA | **GL831156** (3586133-3586195) | 63 | 95.24 | **GL831156** (3586140-3586195) | 56 | 94.64 |
| 128 | TGCAGAGCGTCCAGCAGCTCAAGCACAAACAGATCCTGGTCCAGCTGAGCCAGCGTCGCACGGAGAGAG | **GL831145** (3933020-3933088) | 69 | 91.3 | **GL831145** (3933020-3933088) | 69 | 91.3 |
| 128 | TGCAGAGCGTCCAGCAGCTCAAGCACAAACAGATCCTGGTCCAGCTGAGCCAGCGTCGCACGGAGAGAG | **GL831145** (3933020-3933088) | 69 | 91.3 | **GL831145** (3933020-3933088) | 69 | 91.3 |
| 136 | TGCAGGAGCTGTGTTGTACCGCGTCCCCCTGACCTGGGGTCAGAATAAACTCCCTTGGAAGTTGCTGCA | **GL831199** (3056717-3056777) | 61 | 95.08 | **GL831199** (3056717-3056777) | 61 | 95.08 |
| 139 | TGCAGGTCAACGGCTTCATCCTCATCATCGACTGGAGTAATTTCACCTTCAAGCAGGCGTCCAAACTGA | **GL831427**  (193597-193665) | 69 | 95.65 | **GL831427**  (193597-193665) | 69 | 95.65 |
| 152 | TGCAGCACTCCCAGCTGCCCCTCCTCAGGCCCCATCCTGGGTGGCTCAGTAGTGGGGTAGCAGATGGTG | **GL831138**  (519802-519870) | 69 | 94.20 | **GL831138**  (519801-519870) | 70 | 94.29 |
| 166 | TGCAGAACTGCTGGAGTGAACTCCTCATCCTCGACCACGTCTTCCGGCAGGTGGTGCATG | **GL831204** (1230093-1230152) | 60 | 93.33 | **GL831204** (1230093-1230152) | 60 | 93.33 |
| 204 | TGCAGAAACTGGAGCTGTAACAGGAGATGCCCATCCACTCATCCTACCGCTTCAAATAGGAGGCGAAGG | **GL831177**  (319081-319149) | 69 | 91.3 | **GL831177**  (319079-319150) | 72 | 91.67 |
| 218 | TGCAGACACCGAGATGACCACTCAGATCATCTCCTCCAACCTGGAGCTGCACTCGCTCTCCACAGGCAG | **GL831139** (9052072-9052140) | 69 | 92.75 | **GL831139** (9052072-9052140) | 69 | 92.75 |
| 243 | TGCAGCACTGTACTGGAGGATTAGCAGAATACAAATAGCCCCTCTTGCTCCACCTGCCCTCTACACCGC | **GL831186**  (514300-514364) | 65 | 93.85 | **GL831186**  (514300-514365) | 66 | 93.94 |
| 254 | TGCAGCCACGCCACCAGGACAAATCCCTGCACATTTATTGTACTTAGCAAACAAAGAGGCTGTGAATTA | **GL831135** (10387601-10387669) | 69 | 95.65 | **GL831135** (10387599-10387669) | 71 | 95.77 |
| 257 | TGCAGCCAGGCAGCTCCTTTGTAATGCCAACATGAAATAATCATGACAAGCAGCCCATTGACTGGATCA | **GL831188** (1922084-1922136) | 53 | 98.11 | **GL831188** (1922084-1922136) | 53 | 98.11 |
| 262* | TGCAGCCTCGCAGTCTTTTGGATCCCAAAGTGCTCGGCTTGCAGTCTGGAGGGTGAGTGGTGGCTCACT | **GL831133** (1865353-1865418) | 66 | 93.94 | **GL831133** (1865344-1865419) | 76 | 93.42 |
| 268 | TGCAGCTCAAGTATGTTTGATGCCTGTTCTGGCTCCTCTCTGGTATCTGGGGCAGCGTACCAGCCAGCA | **GL831152** (5391997-5392063) | 67 | 91.04 | **GL831152** (5391997-5392063) | 67 | 91.04 |
| 274 | TGCAGCTCTGGGGAGACAGCCCTGTCAGATGGTTAACAACCACCATTTTTCACACCGCCGCCACTGTCG | **GL831237**  (256653-256717) | 65 | 92.31 | **GL831237**  (256653-256718) | 66 | 92.42 |
| 277 | TGCAGCTGTGTCCGCAGGCGTCATCAGCATGAAACTCTATCCATCCTGCTCGCCCTGCCCACATCCCGT | **GL831145**  (508458-508524) | 67 | 98.51 | **GL831145**  (508458-508524) | 67 | 98.51 |
| 281 | TGCAGCTTCTCTCCCAGGATCTTGTCATCCAGAGTCGTCATGACAACGGCTCACCTGAGGGACGCAGTT | **GL831236**  (659002-659069) | 68 | 92.65 | **GL831236**  (659002-659069) | 68 | 92.65 |
| 285 | TGCAGGAAAAGCACAGGTGTAAATAATAAAATGAATGTTGGCTGAATTCCATTTAGCTGCTTCAGTTTC | **GL831250** (1114510-1114575) | 66 | 93.94 | **GL831250** (1114510-1114575) | 66 | 93.94 |
| 297 | TGCAGGAGGAGCTGCCAAAGCCGAGTGAAGGGCAGAGCAGCACCGGGCACAACCACGCTGAGAGCCTCC | **GL831146** (4894939-4895007) | 69 | 89.86 | **GL831146** (4894939-4895007) | 69 | 89.86 |
| 327 | TGCAGGTGCTGTGCAGCAACAGCGTGCTGGACTCGTCGGAGTACTGGCTGAGGAATGAAAAAGCTCTGT | **GL831147** (6570573-6570639) | 67 | 92.54 | **GL831147** (6570573-6570639) | 67 | 92.54 |
| 352 | TGCAGTGGAAACCGTCATCAAATTAGAATTGAAGTGATTATCCAGGGCAGAGCAGAAGGACAAAATCGA | **GL831282** (1021157-1021220) | 64 | 95.31 | **GL831282** (1021156-1021220) | 65 | 95.38 |
| 358 | TGCAGTGTATTGGTAACTCACGCATCATTCTGTCTCCAGTTGATGGTGCATGTAGCTCCAGTGGGGTCA | **GL831417**  (518693-518746) | 54 | 98.15 | **GL831417**  (518693-518746) | 54 | 98.15 |
| 362 | TGCAGTTATGCAACCCGTTCCCCAGGCTTGGAGGCTTGACCTTTCCGCAAATAACATGTTCATTTCCCT | **GL831330**  (347142-347186) | 45 | 97.78 | **GL831330**  (347142-347187) | 46 | 97.83 |
| 376 | TGCAGATCTTTCTAACGAACAACGCAATGAATATTTTCACGGATACCAGGGGACATGATCACCTTTCAC | **GL831178** (2486238-2486297) | 60 | 93.33 | **GL831178** (2486238-2486297) | 60 | 93.33 |
| 385 | TGCAGGGACTCTAGTTTCTCCTGCTGCTGTTTGATTTTCTCTGCCAGCTCTTTCTGTTTGTCTTCAGCC | **GL831238** (1951493-1951545) | 53 | 96.23 | **GL831238** (1951493-1951545) | 53 | 96.23 |
| 391 | TGCAGTCTGATGGCAACTGAATTATCCAAACACACCAAACCATTACCTCTACCTAACTCAACTTCAGTG | **GL831237** (1569635-1569698) | 64 | 100 | **GL831237** (1569635-1569699) | 65 | 100 |
| 399 | TGCAGCTCTGTTGACGCCATGGAGGAGGCAAAGAAACTGATTGGCACAGGGAACAGGCATCTGGTAATG | **GL831248** (1975754-1975800) | 47 | 97.87 | **GL831248** (1975754-1975800) | 47 | 97.87 |
| 416 | TGCAGACTGGTACGTTTCTGAGGGAGAATGGAGAGTGGGAAATTGTACTGTTTTATTAGAACCCGGTGG | **GL831237** (1265061-1265129) | 69 | 94.2 | **GL831237** (1265061-1265129) | 69 | 94.2 |
| 418 | TGCAGAGGAAGAGGCACATAGGCAACGACATTGTAGCCATCGTGTTCCAGGAGGAGAACACGCCCTTTG | **GL831272** (1690685-1690753) | 69 | 92.75 | **GL831272** (1690685-1690754) | 70 | 92.86 |
| 427** | TGCAGCAGTCGGTGCAGTCGCAGGTTTTCTGAGCTGAAGCTCTGAATCAGTCTGTCACGCTCCTCAACC | **GL831291**  (298118-298182) | 65 | 89.23 | **GL831291**  (298113-298186) | 74 | 89.19 |
| 465 | TGCAGAGCCTCTGACAGGCGGTCCTGGAGCTGCTCGATGCGGCTGTGGTCTTGGACACCGGGACGATCT | **GL831205** (1989982-1990038) | 57 | 94.74 | **GL831205** (1989982-1990038) | 57 | 94.74 |
| 492 | TGCAGCATGACAGCGCTGAGCTCCATGTAAAGGTTGTTAGCCCTCTCCAGTTTCCTCTCATAGTGCTCA | **GL831137** (5302759-5302827) | 69 | 95.65 | **GL831137** (5302759-5302826) | 68 | 95.59 |
| 493 | TGCAGTTTGGTATCCATGGTGATGTGAGGGAAGCTGCCCAGCACAGCCATGACCTCGTCATACTTCTCC | **GL831143**  (533421-533489) | 69 | 97.1 | **GL831143**  (533420-533489) | 70 | 95.71 |
| 498 | TGCAGCTCGGGCATCCCTGAGCCCAGCATCATGGAGAAGAGGTTGATGAAGAGGTTGGCATG | **GL831134** (12227573-12227631) | 59 | 94.92 | **GL831134** (12227573-12227629) | 57 | 94.74 |
| 508 | TGCAGGCCGAGTGTTTGCAAACACCGAGGACTCCTGCTGTCTGCTGGGCATG | **GL831219**  (580535-580582) | 48 | 95.83 | **GL831219**  (580533-580582) | 50 | 96 |
| 516 | TGCAGCCTAAGCTCTCCACACCTGTCCAGTCACCATGGCAACCTCACCGCTGACAGCAAAGAGCTCTGT | **GL831133** (3065626-3065692) | 67 | 94.03 | **GL831133** (3065626-3065692) | 67 | 92.54 |

*262 sequence alignment for GP-H-Genome BLAST

GP-H-Genome: 171475 TCTCTCTGCAGCCTCGCAGTCTTTTGGATCCCAAAGTGCTCGGCTTGCAGTCTGGAGGGTGAGTGGTGGCTCACTT GP-H-Genome: 171550

GL831133.1:1865344 TCTCTCTGAAGCCTCGCAGTCTTTTGGATCCCAAAGTGCTCGGCTTGCAGACCGGGGGGTGAGTGGTAGCTCACTT GL831133.1:1865419

**427 sequence alignment for GP-H-Genome BLAST

GP-H-Genome: 284771 AACCTGCAGCAGTCGGTGCAGTCGCAGGTTTTCTGAGCTGAAGCTCTGAATCAGTCTGTCACGCTCCTCAACCT GP-H-Genome: 284844

GL831291.1:298186 AACCTGCAGCAGTTGGTGCAGTCGCTGGTTTTCAGAACTGAAGCTCTGAATCAGTCGATCTCGCTCCTCCACCT GL831291.1:298113
