# Supplementary material for: Application of DArT seq derived SNP tags for comparative genome analysis in fishes; An alternative pipeline using sequence data from a non-traditional model species, Macquaria ambigua
Source: PLoS One. 2019 Dec 12;14(12):e0226365. doi: 10.1371/journal.pone.0226365 (PMC6907852; doi:10.1371/journal.pone.0226365)
Supplement: S2 Table — Length of the homology varies from 57–72 predominantly 69 base pairs. Calculated score based on E-value, length and percentage of alignment also predominant with Gasterosteus aculeatus suggesting for a closest phylogenetic lineage. (DOCX) [file pone.0226365.s005.docx]

**S2 Table.** **GP-H-Genome fragments (STAGs) with maximum E-value for 17 evolutionarily conserved genes.** length of the homology varies from 57-72 predominantly 69 base pairs. Calculated score based on E-value, length and percentage of alignment also predominant with *Gasterosteus aculeatus* suggesting for a closest phylogenetic lineage.

| No | Length | Sequence | Score | e-value | Identity | Species  (top scored) | Gene |
| --- | --- | --- | --- | --- | --- | --- | --- |
| 1 | 69 | TGCAGAAGTCCGAGATTGTGGATATGGTGAAGAAACACGTGAAGGCCATCACGCTGGCGATAGGCGACG | 105 | 3e-18 | 94.20 | *Oreochromis niloticus* | atp8a2 |
| 2 | 69 | TGCAGATGGGGCAGGTGTTGTTTTCGGAGAGCCAGCGGTCAATGCAGTGGATGTGGAATTCATGGGAGC | 113 | 5e-21 | 95.65 | *Gasterosteus aculeatus* | rnf6 |
| 3 | 69 | TGCAGGTGTGTACATCCTGATTGCGGCGGGGGCTCTGATGATGGTCGTGGGCTTCCTCGGATGCTGCGG | 113 | 5e-21 | 95.65 | *Gasterosteus aculeatus* | cd9b |
| 4 | 69 | TGCAGGTCAACGGCTTCATCCTCATCATCGACTGGAGTAATTTCACCTTCAAGCAGGCGTCCAAACTGA | 113 | 5e-21 | 95.65 | *Gasterosteus aculeatus* | clvs2 |
| 5 | 69 | TGCAGTGTCTCCTCCAGGCCTGGTAGCTGGCTGCTGTCCAGAGTGCAGATGTTACAGGATGCCAGCTTG | 113 | 5e-21 | 95.65 | *Gasterosteus aculeatus* | lyst |
| 6 | 69 | TGCAGATGTGCATGGACTCTGAGAAGCATATGATCTACACGTTTGGCGGTCGCATCCTGACATGTAATG | 105 | 3e-18 | 94.20 | *Oreochromis niloticus* | mkln1 |
| 7 | 71 | CTGCAGAGCCTGGTGGACGACAACATGGTGGACTGCGAGAGAGTGGGTACATCCAACTAC TACTGGGCCTT | 109 | 8e-20 | 94.37 | *Gasterosteus aculeatus* | mnd1 |
| 8 | 68 | CTGCAGGTGATGTACAACCAGTCCACAGCCACCACCTGCTTCCACAGTCTCCCACTGCTGCGCTGTGT | 119 | 9e-23 | 97.06 | *Gasterosteus aculeatus* | piga |
| 9 | 57 | CAGCTCGGGCATCCCTGAGCCCAGCATCATGGAGAAGAGGTTGATGAAGAGGTTGGC | 105 | 1e-18 | 98.25 | *Gasterosteus aculeatus* | pik3ca |
| 10 | 71 | TGCAGACCCATGATCCCAACAAAGAGGCCTTCAAGTGTGAGGAGTGTGGGAAGCACTACA ACACCAAGCTG | 133 | 1e-26 | 98.59 | *Oreochromis niloticus* | plagl2 |
| 11 | 69 | TGCAGTGGCTCAAGTCCGACCGCGCCCTCATGATGCTCTTTAACGACGGCACTTTCCAGGTAAACACAC | 137 | 4e-28 | 100 | *Gasterosteus aculeatus* | plk2b |
| 12 | 67 | TGCAGAAGAAGCAGACCCAGCGCAGCGTCTTCCGCTGTAATGTCTTTGGAGACATTGGCAGCGGCAA | 109 | 2e-19 | 95.52 | *Oreochromis niloticus* | rhot1b |
| 13 | 72 | CCTGCAGTTTGGTATCCATGGTGATGTGAGGGAAGCTGCCCAGCACAGCCATGASCTCGTCATACTTCTCCT | 123 | 1e-23 | 95.83 | *Oreochromis niloticus* | sec63 |
| 14 | 60 | TGCAGTATGGATGTACGGCTTTACAGACAGCCCGAGGAAAGAGGCCTCAGGTAGCCTTGT | 119 | 8e-23 | 100 | *Gasterosteus aculeatus* | sh3pxd2aa |
| 15 | 71 | CTGCAGGCCCCTCCAGACATCTMCCCCGCCAATGTCACCCTGCGCACAGCCAGTGAGACCAGCCTGTGGCT | 121 | 2e-23 | 95.77 | *Gasterosteus aculeatus* | si:ch211-148f13.1 |
| 16 | 67 | TGCAGAGGAAGAGGCACATAGGCAACGACATTGTAGCCATCGTGTTCCAGGAGGAGAACACGCCCTT | 117 | 3e-22 | 97.01 | *Gasterosteus aculeatus* | si:dkey-166d12.2 |
| 17 | 69 | TGCAGCTTATAGCCCATAGAGATGGAGTCATCTGATGTGCTGAGCTCACTGTCTATGGAGGACTGCGAG | 105 | 1e-18 | 94.2 | *Gasterosteus aculeatus* | zgc:66447 |
