# Supplementary material for: Application of DArT seq derived SNP tags for comparative genome analysis in fishes; An alternative pipeline using sequence data from a non-traditional model species, Macquaria ambigua
Source: PLoS One. 2019 Dec 12;14(12):e0226365. doi: 10.1371/journal.pone.0226365 (PMC6907852; doi:10.1371/journal.pone.0226365)
Supplement: S3 Table — Gene atp8a2 and rnf6 are conserved to a specific syntenic block across all species (Chromosome 21 in Gasterosteus aculeatus while in O. niloticus and Gadus morhua consequently in scaffold GL831157 and GeneScaffold 733). (DOCX) [file pone.0226365.s006.docx]

**S3 Table.** **Evolutionary conserved genes with Euteleostomi as a predominant root species.** Gene *atp8a2* and *rnf6* are conserved to a specific syntenic block across all species (Chromosome 21 in *Gasterosteus aculeatus* while in *O. niloticus* and *Gadus morhua* consequently in scaffold GL831157 and GeneScaffold 733).

| **Gene** | **Root (Million years)** | **Root species** | **Specific to** | **Linkage group** | | |
| --- | --- | --- | --- | --- | --- | --- |
|  |  |  |  | Stickleback | Tilapia | Atlantic Cod |
| atp8a2 | 420 | Euteleostomi | vertebrate | Group XXI | GL831157 | GeneScaffold 733 |
| rnf6 | 420 | Euteleostomi | vertebrate | Group XXI | GL831157 | GeneScaffold 733 |
| cd9b* | 420 | Euteleostomi | fish | Group IV | GL831433 | GeneScaffold 337 |
| clvs2 | 550 | Craniata | vertebrate | Group XVIII | GL831427 | GeneScaffold4036 |
| Lyst | 580 | Bilateria | vertebrate | Group VI | GL831145 | GeneScaffold 612 |
| mkln1 | 580 | Bilateria | vertebrate | Group IV | GL831213 | GeneScaffold 2279 |
| mnd1 | 1500 | Fungi/Metazoa | vertebrate | Group IX | GL831212 | GeneScaffold 3852 |
| piga | 1500 | Fungi/Metazoa | vertebrate | Group XVI | GL831258 | GeneScaffold 824 |
| pik3ca** | 550 | Craniata | fish | Group VIII | GL831476 | GeneScaffold 1321 |
| plagl2* | 420 | Euteleostomi | fish | Group XII | GL831199 | GeneScaffold 873 |
| plk2b | 420 | Euteleostomi | vertebrate | Group XIII | GL831176 | GeneScaffold 1288 |
| rhot1b* | 550 | Craniata | fish | Group IX | GL831193 | GeneScaffold 4492 |
| sec63 | 1500 | Fungi/Metazoa | vertebrate | Group XVIII | GL831143 | GeneScaffold 2513 |
| sh3pxd2aa* | 550 | Craniata | fish | Group IX | GL831290 | GeneScaffold 3371 |
| si:ch211-148f13.1* | 550 | Craniata | fish | Group XI | GL831217 | GeneScaffold 1102 |
| si:dkey-166d12.2* | 420 | Euteleostomi | fish | Group XVII | GL831272 | GeneScaffold 179 |
| zgc:66447* | 420 | Euteleostomi | fish | Group IV | GL831174 | GeneScaffold 3590 |

^**^  Duplication event resulting fish specific paralogs.

*** multiple duplication event
